# Evaluation of the STANDARD M10 Flu/RSV/SARS-CoV-2 Fast assay for the detection of influenza A/B viruses, respiratory syncytial virus, and SARS-CoV-2 in nasopharyngeal swab specimens

**DOI:** 10.1128/spectrum.02672-25

**Published:** 2026-01-26

**Authors:** Eunjung Jeong, Minhee Kang, Sun Ae Yun, Tae Yeul Kim, Hee Jae Huh

**Affiliations:** 1 Biomedical Engineering Research Center, Smart Healthcare Research Institute, Samsung Medical Center 36626, Seoul, Republic of Korea; 2 Research Institute for Future Medicine, Samsung Medical Center, Seoul, Republic of Korea; 3 Department of Laboratory Medicine and Genetics, Samsung Medical Center, Sungkyunkwan University School of Medicine 36626, Seoul, Republic of Korea; 4 Department of Medical Device Management and Research, Samsung Advanced Institute for Health Sciences & Technology, Sungkyunkwan University 35017, Seoul, Republic of Korea; Hôpital Saint-Louis, Paris, France

**Keywords:** SARS-CoV-2, influenza, RSV, M10 fast, Allplex, evaluation

## Abstract

The STANDARD M10 Flu/RSV/SARS-CoV-2 Fast assay (M10 Fast), recently CE-marked, is an updated version of the STANDARD M10 Flu/RSV/SARS-CoV-2 assay. It includes additional gene targets to improve sensitivity and coverage and reduces run time from 60 min to 36 min. This study evaluated the analytical and clinical performance of the M10 Fast assay. A total of 664 nasopharyngeal swab specimens (108 influenza A-positive, 119 influenza B-positive, 79 respiratory syncytial virus [RSV]-positive, 165 SARS-CoV-2-positive, 2 influenza A/RSV co-positive, 1 RSV/SARS-CoV-2 co-positive, and 190 negative), confirmed by the BIOFIRE Respiratory Panel 2.1 *plus* (RP2.1*plus*), were tested in parallel using the M10 Fast assay and the Allplex SARS-CoV-2/FluA/FluB/RSV assay (Allplex). RNA standards were serially diluted and tested with the M10 Fast assay to determine the limit of detection (LOD). Using RP2.1*plus* as the reference standard, the M10 Fast assay demonstrated sensitivities of 98.2%, 100%, 95.1%, and 100% for influenza A, influenza B, RSV, and SARS-CoV-2, respectively. The Allplex assay showed sensitivities of 88.2%, 100%, 91.5%, and 100% for the same viruses. Both assays exhibited high specificity (>99%). The LODs of the M10 Fast assay were 7.8, 35.3, 532.7, 34.7, and 88.0 copies/mL for influenza A, influenza B, RSV, and SARS-CoV-2 ORF1ab/E and N genes, respectively, and were lower than previously reported Allplex LODs, except for RSV. The M10 Fast assay reliably detects influenza A and B, RSV, and SARS-CoV-2 with high sensitivity and specificity, demonstrating performance comparable to or exceeding that of the widely used Allplex assay.

**IMPORTANCE:**

The STANDARD M10 Flu/RSV/SARS-CoV-2 Fast assay (M10 Fast) is an updated version of the STANDARD M10 Flu/RSV/SARS-CoV-2 assay. This study demonstrates that the M10 Fast assay reliably detects influenza A and B, respiratory syncytial virus (RSV), and SARS-CoV-2 with high sensitivity and specificity, including improved detection at low viral loads. Its enhanced performance and shorter turnaround time support timely clinical decision-making and strengthen public health responses, particularly during periods of cocirculation of influenza, RSV, and SARS-CoV-2.

## INTRODUCTION

The cocirculation of severe acute respiratory syndrome coronavirus 2 (SARS-CoV-2) with other respiratory viruses, such as influenza A/B and respiratory syncytial virus (RSV), poses significant challenges to healthcare providers and clinical microbiology laboratories. As these viruses cause similar symptoms and signs, clinical differentiation is challenging (1–3). Further complicating matters, co-infections can occur, although their clinical significance remains unclear (4–6). Therefore, assays capable of simultaneously detecting and differentiating multiple respiratory viruses are needed.

Traditionally, central laboratory-based multiplex molecular assays have been considered the gold standard for simultaneously detecting and differentiating multiple respiratory viruses (7). However, fully automated multiplex molecular assays suitable for point-of-care (POC) use—such as Xpert Xpress SARS-CoV-2/Flu/RSV (Xpert) and Xpert Xpress CoV-2/Flu/RSV *plus* (Xpert *plus*; both Cepheid, Sunnyvale, CA, USA), and BIOFIRE Respiratory Panel 2.1 *plus* (RP2.1*plus*; bioMérieux, Marcy l’Étoile, France)—have recently emerged as attractive alternatives due to their rapid turnaround times and robust performance (8–13). The STANDARD M10 Flu/RSV/SARS-CoV-2 Fast assay (M10 Fast; SD Biosensor, Suwon, Korea), CE-marked in August 2025, is a fully automated multiplex molecular assay suitable for POC use and an updated version of the STANDARD M10 Flu/RSV/SARS-CoV-2 assay (M10; SD Biosensor). Overall, the M10 assay demonstrated performance comparable to widely used molecular assays, such as the Xpert assay, but had limited ability to detect low viral loads (14–16). The M10 Fast assay incorporates additional gene targets to improve sensitivity and coverage while reducing run time from 60 to 36 min; however, performance data remain limited.

This study assessed the analytical and clinical performance of the M10 Fast assay and compared it with the Allplex SARS-CoV-2/FluA/FluB/RSV assay (Allplex; Seegene, Seoul, Korea), a widely used central laboratory-based molecular assay.

## MATERIALS AND METHODS

### Clinical specimens

This study included 664 nasopharyngeal swab specimens in viral transport medium (VTM): 545 collected as part of routine patient care at Samsung Medical Center, Seoul, Korea, between February 2023 and February 2025 (108 influenza A-positive, 79 RSV-positive, 165 SARS-CoV-2-positive, 2 influenza A/RSV co-positive, 1 RSV/SARS-CoV-2 co-positive, and 190 negative), and 119 influenza B-positive specimens obtained from Trina Bioreactives AG, Nänikon, Switzerland. All specimens were confirmed using RP2.1 plus and stored at −70°C until use. After thawing at room temperature, they were tested simultaneously with the M10 Fast and Allplex assays by two different technicians blinded to specimen identities and prior test results.

### Molecular assays

RP2.1*plus*, a fully automated multiplex molecular assay capable of simultaneously detecting 23 respiratory pathogens—including SARS-CoV-2, influenza A/B, and RSV—within 45 min, was performed according to the manufacturer’s instructions. Briefly, 300 µL of specimen was mixed with the sample buffer and loaded into the test pouch, which was inserted into the BioFire FilmArray 2.0 instrument, where nucleic acid extraction, amplification, and result analysis were performed automatically.

The M10 Fast assay, targeting the ORF1ab, E, and N genes of SARS-CoV-2; the M and PB2 genes of influenza A; the NS1 and M genes of influenza B; and the M and N genes of RSV, was performed according to the manufacturer’s instructions. Briefly, 300 µL of specimen was loaded into the test cartridge and inserted into the STANDARD M10 instrument, where nucleic acid extraction, amplification, and result analysis were performed automatically.

The Allplex assay, targeting the RdRp, S, and N genes of SARS-CoV-2; the M gene of influenza A; the NS2 gene of influenza B; and the M gene of RSV, was performed according to the manufacturer’s instructions. In brief, RNA was extracted using the KingFisher Flex (Thermo Fisher Scientific, Waltham, MA, USA), and 10 µL of extracted RNA was combined with 10 µL of reaction master mix to yield a final volume of 20 µL. Amplification was performed on a CFX96 Real-Time PCR Detection System (Bio-Rad, Hercules, CA, USA) under the following cycling conditions: 50°C for 20 min, 95°C for 15 min, 3 cycles of 95°C for 10 s, 60°C for 40 s, and 72°C for 20 s, followed by 42 cycles of 95°C for 10 s, 60°C for 15 s, and 72°C for 10 s. Results were automatically analyzed using Seegene Viewer software.

For specimens with discordant results, further analysis was performed using the cobas Liat SARS-CoV-2 and Influenza A/B assay or the cobas Liat Influenza A/B & RSV assay (Roche Diagnostics, Basel, Switzerland), fully automated molecular assays providing results within 20 min. These assays, hereafter collectively referred to as cobas Liat, were performed according to the manufacturer’s instructions. Briefly, 200 µL of specimen was loaded into the assay tube and inserted into the cobas Liat Analyzer, where nucleic acid extraction, amplification, and result analysis were performed automatically. 

### Analytical performance evaluation

The limits of detection (LODs) and reproducibility of the M10 Fast assay were evaluated using the AMPLIRUN TOTAL SARS-CoV-2/FluA/FluB/RSV CONTROL (Vircell, Granada, Spain). This RNA standard was serially diluted in a pool of negative nasopharyngeal swab specimens in VTM and tested in 20 replicates per dilution level. Cross-reactivity of the M10 Fast assay was assessed against 28 respiratory pathogens not targeted by the assay, with each pathogen tested in duplicate at a clinically relevant concentration. Specifically, bacteria and yeast were tested at ≥1 × 10⁶ CFU/mL or copies/mL, while viruses were tested at ≥1 × 10⁵ copies/mL.

### Data analysis

Sensitivity and specificity of the M10 Fast and Allplex assays were determined using RP2.1*plus* as the reference standard. Positive percent agreement (PPA), negative percent agreement (NPA), and Cohen’s kappa between the two assays were calculated. Ct values in specimens positive by both assays were compared using Pearson’s correlation coefficient (*r*). LODs were determined by probit regression analysis. Reproducibility was assessed by calculating the coefficients of variation (CV) of Ct values. All statistical analyses were performed using Excel (Microsoft, Redmond, WA, USA) and MedCalc Statistical Software version 23.0.6 (MedCalc Software Ltd., Ostend, Belgium).

## RESULTS

### Clinical performance

**Fig 1:**
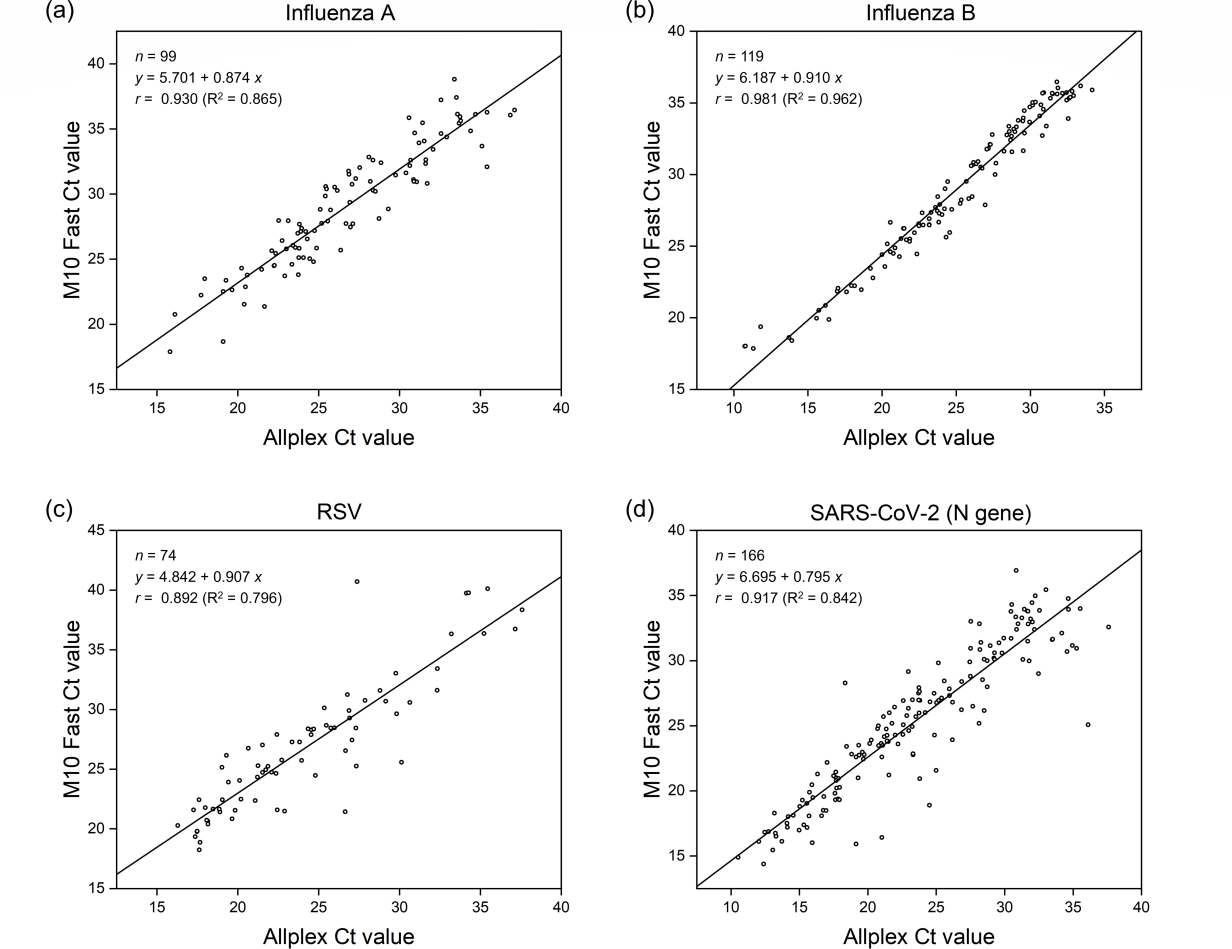
Correlation of Ct values for each target between the M10 Fast and Allplex assays: (**a**) influenza A; (**b**) influenza B; (**c**) RSV; (**d**) SARS-CoV-2 (N gene).

Using RP2.1*plus* as the reference standard, the M10 Fast assay demonstrated sensitivities of 98.2%, 100%, 95.1%, and 100% for influenza A, influenza B, RSV, and SARS-CoV-2, respectively, whereas the Allplex assay showed sensitivities of 88.2%, 100%, 91.5%, and 100% for the same targets. Both assays exhibited high specificity (>99%) for all viruses (Table 1). Agreement between the two assays was high, with PPA ranging from 98.0% to 100% and NPA from 97.9% to 100%. Cohen’s kappa values ranged from 0.92 to 1.00, indicating almost perfect agreement (Table S1). Among the three co-infection specimens confirmed by RP2.1*plus*, the M10 Fast assay detected all corresponding targets in all specimens, whereas the Allplex assay missed influenza A in one influenza A/RSV co-positive specimen. Ct values demonstrated a strong linear relationship across all viral targets, with Pearson’s correlation coefficient (*r*) ranging from 0.892 to 0.981 (Fig. 1).

**TABLE 1:** Clinical performance comparison of the M10 fast and Allplex assays^*a*^

Viral target	No. of TP	No. of FN	No. of TN	No. of FP	Sensitivity (95% CI)	Specificity (95% CI)
M10 Fast assay						
Influenza A	108	2	551	3	98.2% (93.6%–99.8%)	99.5% (98.4%–99.9%)
Influenza B	119	0	545	0	100% (96.9%–100%)	100% (99.3%–100%)
RSV	78	4	581	1	95.1% (88.0%–98.7%)	99.8% (99.0%–100%)
SARS-CoV-2	166	0	494	4	100% (97.8%–100%)	99.2% (98.0%–99.8%)
Allplex assay						
Influenza A	97	13	550	4	88.2% (80.6%–93.6%)	99.3% (98.2%–99.8%)
Influenza B	119	0	545	0	100% (96.9%–100%)	100% (99.3%–100%)
RSV	75	7	582	0	91.5% (83.2–96.5%)	100% (99.4–100%)
SARS-CoV-2	166	0	498	0	100% (97.8–100%)	100% (99.3–100%)

^*a*^ TP, true positives; FN, false negatives; TN, true negatives; FP, false positives; CI, confidence interval.

### Analysis of discordant results

A total of 31 specimens showed discordant results between RP2.1*plus* and the M10 Fast or Allplex assays, as detailed in Table S2. All had high Ct values for the corresponding targets (>30). The most frequent discordances involved influenza A (*n* = 18). Of these, 13 were confirmed as influenza A-positive by RP2.1*plus* but tested negative in the Allplex assay alone (*n* = 11) or in both the M10 Fast and Allplex assays (*n* = 2). When further analyzed using cobas Liat, 11 of these 13 specimens were influenza A-positive, while two were negative. The remaining five were influenza A-negative by RP2.1*plus* but tested positive in the M10 Fast assay alone (*n* = 1), Allplex assay alone (*n* = 2), or both assays (*n* = 2); only the latter two were confirmed as influenza A–positive by cobas Liat.

The second most frequent discordances involved RSV (*n* = 9). Of these, eight were confirmed as RSV-positive by RP2.1*plus* but tested negative in the M10 Fast assay alone (*n* = 1), Allplex assay alone (*n* = 4), or both assays (*n* = 3). When further analyzed using cobas Liat, seven of these eight specimens were RSV–positive, while one was negative. The remaining specimen, RSV-negative by RP2.1*plus* but positive in the M10 Fast assay alone, was confirmed as RSV-positive by cobas Liat.

The remaining discordances involved SARS-CoV-2 (*n* = 4), all of which were confirmed as SARS-CoV-2-negative by RP2.1*plus* but positive in the M10 Fast assay alone; three were SARS-CoV-2-negative, and one was positive by cobas Liat.

### Analytical performance

The LODs of the M10 Fast assay were 7.8, 35.3, 532.7, 34.7, and 88.0 copies/mL for influenza A, influenza B, RSV, and the SARS-CoV-2 ORF1ab/E and N genes, respectively. The assay demonstrated high inter-run reproducibility, with Ct CVs ≤3.5% at concentrations above 2× LOD (Table 2). In the analytical specificity evaluation, no cross-reactivity was observed with any of the tested respiratory pathogens (Table 3).

**TABLE 2:** Analytical sensitivity and reproducibility evaluation results of the M10 fast assay^*a*^

Concentration (copies/mL)	Influenza A			Influenza B			RSV			SARS-CoV-2					
										ORF1ab/E gene			N gene		
	n/N (%)	Mean Ct	Ct CV%	n/N (%)	Mean Ct	Ct CV%	n/N (%)	Mean Ct	Ct CV%	N/N (%)	Mean Ct	Ct CV%	n/N (%)	Mean Ct	Ct CV%
2000	20/20 (100)	33.7	1.2	20/20 (100)	35.2	1.3	20/20 (100)	38.5	1.2	20/20 (100)	35.2	1.6	20/20 (100)	35.5	1.5
800	20/20 (100)	34.7	1.0	20/20 (100)	36.1	1.4	19/20 (95)	39.6	2.0	20/20 (100)	36.4	1.6	20/20 (100)	36.5	1.6
400	20/20 (100)	36.1	2.0	20/20 (100)	37.5	1.6	17/20 (85)	40.0	1.6	20/20 (100)	37.7	1.2	20/20 (100)	38.0	1.6
200	20/20 (100)	37.3	3.0	20/20 (100)	38.6	2.5	16/20 (80)	41.0	1.9	20/20 (100)	38.5	2.0	20/20 (100)	38.8	2.2
100	20/20 (100)	38.1	2.5	20/20 (100)	38.9	3.5	7/20 (35)	40.8	0.7	20/20 (100)	39.4	1.9	19/20 (95)	39.4	2.5
20	20/20 (100)	39.0	1.8	10/20 (50)	40.3	1.3	3/20 (15)	41.3	1.4	13/20 (65)	40.3	2.2	11/20 (55)	40.6	2.4
5	16/20 (80)	40.1	1.6	2/20 (10)	40.1	1.7	0/20 (0)	N/A	N/A	5/20 (25)	41.1	1.3	1/20 (5)	39.8	N/A
2.5	11/20 (55)	40.5	0.9	0/20 (0)	N/A	N/A	0/20 (0)	N/A	N/A	2/20 (10)	40.4	1.2	1/20 (5)	39.8	N/A
Probit LoD (95% CI)	7.8 (6.0–9.6)			35.3 (27.3–58.7)			532.7 (466.8–598.7)			34.7 (25.5–63.3)			88.0 (64.9–143.0)		

^*a*^ n/N, no. detected/no. of replicates; Ct, cycle threshold; CV, coefficient of variation; N/A, not applicable; LoD, limit of detection; CI, confidence interval.

**TABLE 3:** Analytical specificity evaluation of the M10 fast assay^*a*^

Organism	Source (code no.)	Result
MERS-CoV	Vircell (MBC132)	Negative
Human coronavirus 229E	ATCC (VR-740D)	Negative
Human coronavirus OC43	Vircell (MBC135-R)	Negative
Human coronavirus NL63	Vircell (MBC142-R)	Negative
Human coronavirus HKU1	ATCC (VR-3262SD)	Negative
Human parainfluenza virus 1	Vircell (MBC037)	Negative
Human parainfluenza virus 2	Vircell (MBC038)	Negative
Human parainfluenza virus 3	Vircell (MBC039)	Negative
Human parainfluenza virus 4	Vircell (MBC050)	Negative
Enterovirus D68	Vircell (MBC125)	Negative
Rhinovirus B14	Vircell (MBC091)	Negative
Human adenovirus 1	Vircell (MBC001)	Negative
Human metapneumovirus	KBPV (VR-87D)	Negative
Cytomegalovirus	NIBSC (09/162)	Negative
*Streptococcus pneumoniae*	ATCC (33400D-5)	Negative
*Haemophilus influenzae*	ATCC (51907D-5)	Negative
*Chlamydophila pneumoniae*	ATCC (53592D)	Negative
*Mycoplasma pneumoniae*	ATCC (15531D)	Negative
*Legionella pneumophila*	ATCC (33152D-5)	Negative
*Bordetella pertussis*	ATCC (9797D-5)	Negative
*Bordetella parapertussis*	ATCC (15311D-5)	Negative
*Staphylococcus aureus*	ATCC (29213)	Negative
*Staphylococcus epidermidis*	ATCC (12228)	Negative
*Streptococcus pyogenes*	ATCC (19615)	Negative
*Pseudomonas aeruginosa*	ATCC (27853)	Negative
*Neisseria meningitidis*	ATCC (13100)	Negative
*Escherichia coli*	ATCC (25922)	Negative
*Candida albicans*	ATCC (90028)	Negative

^*a*^ MERS-CoV, Middle East respiratory syndrome coronavirus; ATCC, American Type Culture Collection; KBPV, Korea Bank for Pathogenic Viruses; NIBSC, The National Institute for Biological Standards and Control. Each organism was tested at clinically relevant concentrations: ≥1 × 10⁶ CFU/mL or ≥1 × 10⁶ copies/mL for bacteria and yeasts, and ≥1 × 10⁵ copies/mL for viruses.

## DISCUSSION

In this study, we evaluated the analytical and clinical performance of the M10 Fast assay, an updated version of the M10 assay, and compared it with the Allplex assay, a widely used commercial molecular test. Using RP2.1*plus* as the reference standard, the M10 Fast assay demonstrated high sensitivity (>95%) and specificity (>99%) for all viral targets, comparable to those of the Allplex assay. The M10 Fast assay demonstrated high analytical sensitivity, with LODs <100 copies/mL for all targets, except RSV (532.7 copies/mL). These results indicate that the M10 Fast assay is highly sensitive and accurate for detecting influenza A/B, RSV, and SARS-CoV-2. Given its high accuracy, rapid turnaround time (36 min), and fully automated, cartridge-based format, the M10 Fast assay is a valuable tool for detecting these viruses in POC settings.

A key feature of the M10 Fast assay, compared with the previous M10 assay, is the incorporation of an additional gene target for each virus (PB2 for influenza A, M for influenza B, N for RSV, and E for SARS-CoV-2) to improve sensitivity and coverage. As expected, its analytical sensitivity was excellent, with LODs of 7.8, 35.3, 532.7, 34.7, and 88.0 copies/mL for influenza A, influenza B, RSV, and SARS-CoV-2 ORF1ab/E and N genes, respectively—substantially lower than those reported for the M10 assay (15). These values were also considerably lower than those of the Allplex assay, except for RSV (17), and comparable to those of the Xpert *plus* assay, which is known to have superior sensitivity compared with the M10 assay (15, 16). This enhanced sensitivity enabled the M10 Fast assay to detect more influenza A-positive and RSV-positive specimens confirmed by RP2.1*plus* than the Allplex assay. Although the M10 Fast assay failed to detect two influenza A-positive and four RSV-positive specimens, all but one were also missed by the Allplex assay. Further analysis with cobas Liat showed that these specimens were either not detected or detected with Ct values >30, suggesting low viral loads. Notably, the M10 Fast assay detected influenza A (*n* = 3), RSV (*n* = 1), or SARS-CoV-2 (*n* = 4) in eight specimens that tested negative for the corresponding targets by RP2.1*plus*. While the results from these specimens were classified as false positives in our study, some were also detected by the Allplex assay or cobas Liat, suggesting they may reflect the high sensitivity of the M10 Fast assay. Importantly, the M10 Fast assay incorporates two to three gene targets per virus, unlike the Allplex and cobas Liat assays, which use a single target per virus (except SARS-CoV-2). This multi-target design minimizes the impact of viral genetic mutations on assay sensitivity. Further confirmation using more sensitive molecular methods is needed to determine whether these results represent true or false positives.

The M10 Fast assay demonstrated overall good performance in this study; however, its failure to detect two influenza A-positive specimens and four RSV-positive specimens identified by RP2.1*plus* raises concern. The relatively low sensitivity for RSV may be attributable to the high LOD for this target. Because some patients with clinically significant RSV infection can have viral loads as low as 10²–10³ copies/mL in nasopharyngeal specimens (18, 19), the inability to reliably detect viral loads in this range represents a significant limitation. While the manufacturer has not disclosed the exact cause of the high LOD, assay design factors—such as the selected target region or primer/probe configuration—may reduce amplification efficiency at low viral concentrations. Failure to detect virus-positive specimens may also result from sequence variations in the primer/probe regions. Respiratory viruses, including SARS-CoV-2 and influenza, mutate rapidly enough to occasionally affect the performance of real-time PCR assays (20, 21); however, given the multi-target design of the M10 Fast assay, missing virus-positive specimens due to sequence variations is unlikely. The Xpert *plus* assay employs a similar multi-target design, and comparative evaluation of the two assays will be a focus of future studies.

Fully automated, cartridge-based assays, such as M10 Fast, Xpert, RP2.1*plus*, and cobas Liat, offer several advantages. They integrate sample preparation, nucleic acid extraction, amplification, and detection into a single workflow, reducing turnaround time, minimizing manual handling, and lowering the risk of human error. All reactions occur within a closed cartridge, further minimizing the risk of cross-contamination. The instruments used for these assays are compact and can operate outside central laboratories, such as in outpatient clinics or emergency rooms, making them suitable for POC use (22, 23). Despite these benefits, these assays are generally more expensive than central laboratory-based molecular tests, such as the Allplex assay, which may limit their use in low-resource settings. They may also be less suitable for meeting high testing demand during a pandemic due to limited throughput (24, 25). Although throughput can be scaled up by installing additional modules, this entails considerable cost (23). Assay cost remains a major barrier to the widespread adoption of fully automated, cartridge-based assays in low-resource settings; however, their rapid turnaround time can reduce the length of hospital stay and decrease unnecessary antibiotic use (26, 27), and the resulting cost savings may offset the higher assay cost. Clinical microbiology laboratories should select molecular assays based not only on testing demands and available resources but also on the potential for such cost savings.

One major limitation of this study is the use of archived specimens. Virus-positive specimens confirmed by RP2.1*plus* may have undergone RNA degradation during storage and freeze–thaw cycles, potentially underestimating the clinical sensitivity of the M10 Fast and Allplex assays. However, because the two assays were tested in parallel, this effect likely influenced them to a similar degree. Furthermore, our specimens underwent only a single freeze–thaw cycle, and previous studies conducted in similar settings have demonstrated that detection of respiratory viruses by real-time PCR is not significantly affected by long-term storage of specimens (25, 28–30). Therefore, we believe that RNA degradation is unlikely to be a major factor contributing to false-negative results in the M10 Fast and Allplex assays. To more accurately assess assay performance, particularly for specimens with low viral loads, prospective studies using fresh specimens are needed. Another limitation is that influenza B-positive specimens were sourced externally, and heterogeneity in sample collection, storage, and transport may have affected assay performance. However, the high performance observed for influenza B is likely attributable to the absence of low viral load specimens (Ct values >37), and further studies, including such specimens are needed. Additionally, only three co-infection specimens were included, which is insufficient to evaluate the assay’s ability to detect co-infections. Finally, the single-center design limits the generalizability of our findings, highlighting the need for multicenter evaluations.

In conclusion, our findings demonstrate that the updated M10 Fast assay reliably detects influenza A/B, RSV, and SARS-CoV-2 with high sensitivity and specificity, offering improved detection of low viral loads. Its enhanced performance and faster turnaround time support timely clinical decision-making and public health responses, particularly during periods of influenza, RSV, and SARS-CoV-2 cocirculation.

## SUPPLEMENTAL MATERIAL

Tables S1 and S2 spectrum.02672-25-s0001.docx Table S1: Concordance analysis between the M10 Fast and Allplex assays. Table S2: Details of discordant results.

ASM does not own the copyrights to Supplemental Material that may be linked to, or accessed through, an article. The authors have granted ASM a non-exclusive, world-wide license to publish the Supplemental Material files. Please contact the corresponding author directly for reuse.
