# Supplementary material for: Evaluation of the STANDARD M10 Flu/RSV/SARS-CoV-2 Fast assay for the detection of influenza A/B viruses, respiratory syncytial virus, and SARS-CoV-2 in nasopharyngeal swab specimens
Source: Microbiol Spectr. 2026 Jan 26;14(3):e02672-25. doi: 10.1128/spectrum.02672-25 (PMC12955378; doi:10.1128/spectrum.02672-25)
Supplement: Tables S1 and S2 — Table S1: Concordance analysis between the M10 Fast and Allplex assays. Table S2: Details of discordant results. [file spectrum.02672-25-s0001.docx]

**Table S1.** Concordance analysis between the M10 Fast and Allplex assays

| Viral target | | M10 Fast result | Allplex result | | PPA  (95% CI) | NPA  (95% CI) | Kappa value  (95% CI) |
| --- | --- | --- | --- | --- | --- | --- | --- |
|  |  |  | Positive | Negative |  |  |  |
| Influenza A | Positive | | 99 | 12 | 98.0%  (93.0–99.8%) | 97.9%  (96.3–98.9%) | 0.92  (0.88–0.96) |
|  | Negative | | 2 | 551 |  |  |  |
| Influenza B | Positive | | 119 | 0 | 100%  (96.9–100%) | 100%  (99.3–100%) | 1.00  (1.00–1.00) |
|  | Negative | | 0 | 545 |  |  |  |
| RSV | Positive | | 74 | 5 | 98.7%  (92.8–100%) | 99.2%  (98.0–99.7%) | 0.96  (0.92–0.99) |
|  | Negative | | 1 | 584 |  |  |  |
| SARS-CoV-2 | Positive | | 166 | 4 | 100%  (97.8–100%) | 99.2%  (98.0–99.8%) | 0.98  (0.97–1.00) |
|  | Negative | | 0 | 494 |  |  |  |

PPA, positive percent agreement; NPA, negative percent agreement; CI, confidence interval.

**Table S2.** Details of discordant results

| Specimen no. | Reference |  | Comparative testing | | |  | Discordant testing |
| --- | --- | --- | --- | --- | --- | --- | --- |
|  | RP2.1*plus* |  | M10 Fast |  | Allplex |  | cobas Liat |
|  | Detected target |  | Detected target (Ct value) |  | Detected target (Ct value) |  | Detected target (Ct value) |
| **Specimens with discordant results for Flu A (*n* = 18)** | | | | | | | |
| 91 | Flu A |  | Flu A (37.0) |  | None |  | Flu A (29.5) |
| 124 | Flu A |  | Flu A (37.8) |  | None |  | Flu A (32.4) |
| 237 | Flu A |  | Flu A (33.0) |  | None |  | Flu A (25.2) |
| 330 | Flu A |  | Flu A (38.8) |  | None |  | Flu A (31.6) |
| 351 | Flu A |  | Flu A (36.7) |  | None |  | Flu A (28.9) |
| 389 | Flu A |  | Flu A (39.6) |  | None |  | Flu A (32.5) |
| 413 | Flu A |  | Flu A (37.2) |  | None |  | Flu A (28.9) |
| 568 | Flu A |  | Flu A (38.4) |  | None |  | Flu A (34.1) |
| 625 | Flu A |  | Flu A (36.8) |  | None |  | Flu A (27.3) |
| 243 | Flu A, RSV |  | Flu A (39.5), RSV (31.6) |  | RSV (32.3) |  | Flu A (34.4), RSV (23.6) |
| 368 | Flu A |  | Flu A (40.9) |  | None |  | None |
| 261 | Flu A |  | None |  | None |  | None |
| 465 | Flu A |  | None |  | None |  | Flu A (32.9) |
| 214 | Flu B |  | Flu A (40.7), Flu B (30.0) |  | Flu B (27.6) |  | Flu B (22.0) |
| 424 | Flu B |  | Flu B (26.5) |  | Flu A (34.8), Flu B (23.2) |  | Flu B (17.3) |
| 456 | SARS-CoV-2 |  | SARS-CoV-2 (28.8, 28.4) |  | Flu A (34.5), SARS-CoV-2 (24.9, 23.5, 25.6) |  | SARS-CoV-2 (21.4) |
| 221 | RSV |  | Flu A (36.1), RSV (30.8) |  | Flu A (34.7), RSV (27.9) |  | Flu A (29.3), RSV (24.0) |
| 359 | RSV |  | Flu A (33.7), RSV (17.3) |  | Flu A (35.9), RSV (27.9) |  | Flu A (34.0), RSV (14.0) |
| **Specimens with discordant results for RSV (*n* = 9)** | | | | | | | |
| 300 | RSV |  | None |  | RSV (36.4) |  | RSV (33.5) |
| 242 | RSV |  | RSV (35.7) |  | None |  | RSV (30.7) |
| 253 | RSV |  | RSV (36.2) |  | None |  | RSV (31.3) |
| 395 | RSV |  | RSV (39.8) |  | None |  | RSV (33.4) |
| 410 | RSV |  | RSV (40.9) |  | None |  | RSV (30.5) |
| 130 | RSV |  | None |  | None |  | RSV (35.1) |
| 584 | RSV |  | None |  | None |  | RSV (33.9) |
| 250 | RSV |  | None |  | None |  | None |
| 381 | None |  | RSV (41.0) |  | None |  | RSV (34.7) |
| **Specimens with discordant results for SARS-CoV-2 (*n* = 4)** | | | | | | | |
| 281 | Flu A |  | Flu A (27.1), SARS-CoV-2 (−, 38.9) |  | Flu A (24.2) |  | Flu A (22.3) |
| 365 | Flu A |  | Flu A (26.1), SARS-CoV-2 (41.3, −) |  | Flu A (23.4) |  | Flu A (22.4) |
| 552 | Flu B |  | Flu B (25.3), SARS-CoV-2 (−, 39.4) |  | Flu B (21.9) |  | Flu B (16.1) |
| 595 | Flu A |  | Flu A (31.2), SARS-CoV-2 (−, 40.4) |  | Flu A (27.3) |  | Flu A (24.7), SARS-CoV-2 (37.3) |

Flu A, influenza A; Flu B, influenza B. For SARS-CoV-2, Ct values are shown in the following order: M10 Fast assay (ORF1ab/E, N) and Allplex assay (RdRp, S, N).
